# Identification of key regulatory genes and their working mechanisms in type 1 diabetes

**DOI:** 10.1186/s12920-023-01432-y

**Published:** 2023-01-17

**Authors:** Hui Li, Xiao Hu, Jieqiong Li, Wen Jiang, Li Wang, Xin Tan

**Affiliations:** Pediatric Department, The First Hospital of Changsha, No. 311, Yingpan Road, Kaifu District, Changsha, 410000 Hunan People’s Republic of China

**Keywords:** Type 1 diabetes, WGCNA, GEO, Differentially expressed genes

## Abstract

**Background:**

Type 1 diabetes (T1D) is an autoimmune disease characterized by the destruction of beta cells in pancreatic islets. Identification of the key genes involved in T1D progression and their mechanisms of action may contribute to a better understanding of T1D.

**Methods:**

The microarray profile of T1D-related gene expression was searched using the Gene Expression Omnibus (GEO) database. Then, the expression data of two messenger RNAs (mRNAs) were integrated for Weighted Gene Co-Expression Network Analysis (WGCNA) to generate candidate genes related to T1D. In parallel, T1D microRNA (miRNA) data were analyzed to screen for possible regulatory miRNAs and their target genes. An miRNA–mRNA regulatory network was then established to predict the key regulatory genes and their mechanisms.

**Results:**

A total of 24 modules (i.e., clusters/communities) were selected using WGCNA analysis, in which three modules were significantly associated with T1D. Further correlation analysis of the gene module revealed 926 differentially expressed genes (DEGs), of which 327 genes were correlated with T1D. Analysis of the miRNA microarray showed that 13 miRNAs had significant expression differences in T1D. An miRNA–mRNA network was established based on the prediction of miRNA target genes and the combined analysis of mRNA, in which the target genes of two miRNAs were found in T1D correlated genes.

**Conclusion:**

An miRNA–mRNA network for T1D was established, based on which 2 miRNAs and 12 mRNAs were screened, suggesting that they may play key regulatory roles in the initiation and development of T1D.

**Supplementary Information:**

The online version contains supplementary material available at 10.1186/s12920-023-01432-y.

## Introduction

The diagnosis of type 1 diabetes (T1D) is based on clinical manifestations of insulin-dependent diabetes, and the majority of cases are found in childhood or puberty with diabetic ketoacidosis [1]. Furthermore, data has shown that the incidence of T1D in children is still increasing [2, 3]. Traditionally, T1D is considered an immune disease caused by the destruction of pancreatic beta cells by T lymphocytes, leading to hyperglycemia and hypoglycemia-related complications [4]. Local islet inflammation can be partially attributed to the regulation ofbeta cells and infiltrating immune cells. In addition, increasing evidence has shown that islet inflammation can be ascribed to T1D candidate genes and environmental factors, including viral infection [5–8]. Research exploring the regulatory mechanism of T1D showed that miR-92a could regulate *KLF2* expression to influence the function of B cells [9]. In addition, genes such as *RNASEH1*, *BANK1*, and *SLC40A1* have also been reported to be involved in T1D regulation [10–12]. Nevertheless, data concerning key regulatory genes in T1D remain to be clarified, and more effort is required before a clearer understanding of T1D pathogenesis can be acquired. Therefore, screening for T1D regulatory genes may provide new directions for a better understanding of T1D pathology.

Weighted correlation network analysis (WGCNA) is a data mining (or computational) method used to describe correlation patterns among genes through gene co-expression networks in complicated diseases [13]. WGCNA has been used successfully to identify disease-related modules (clusters/communities) and genes. For instance, Chen et al. highlighted two modules and genes associated with bone mineral density through WGCNA [14, 15]. Riquelme et al*.* compared the data of T1D patients and healthy controls in one microarray using WGCNA and identified modules in co-expression gene networks that may be associated with T1D, in which the possible regulatory genes in T1D were identified [16]. The majority of previous studies mainly focused on single susceptibility genes or genome-wide association studies to explore the possible hereditary factors for T1D [17–20].

The importance of the immune system in the development of diabetes was reported in a previous study [21], and further evidence indicated the involvement of peripheral blood mononuclear cells (PBMCs) in the immunoregulation of T1D [22]. To broaden the data size, we integrated data from the T1D PBMC microarray to enable data excavation on T1D regulatory genes using T1D as a relative character. The integrated data were then subjected to WGCNA analysis to determine the possible regulatory genes. In a study by Roggli et al., some microRNAs (miRNAs), including miR-21 and miR-34a, were possibly involved in beta cell apoptosis in diabetes [23]. In addition, let-7c-5p and miR-25 have been reported to serve as biomarkers for T1D initiation and development [24–27]. Thisevidence supports the possible regulatory role of miRNAs in T1D. In this study, we used WGCNA analysis to analyze the T1D miRNA microarray to identify the key regulatory genes, and explored their possible working mechanisms in T1D after conjoint analysis on the T1D miRNA microarray. This study aimed to provide a potential therapeutic basis for a better understanding of the initiation and development of T1D.

## Materials and methods

### Data download and treatment

T1D microarray data were screened in the Gene Expression Omnibus (GEO) database (https://www.ncbi.nlm.nih.gov/geo/) based on the criteria that the sample size for both controls and T1D should be greater than 10, and the sample source should be PBMCs. Three independent T1D microarrays were obtained, including two messenger RNA (mRNA) microarrays and one miRNA microarray (Table 1) [28, 29]. Then, the two mRNA microarrays were integrated using the R limma package and sva package [30–32] and subjected to Bayesian adjustments. Genes co-expressed in both microarrays were saved during integration.

**Table 1:** Data downloaded from GEO database for analysis

GEO catalog	microarrays	Sample size for healthy controls	Sample size for T1D	Platform	Sample type
GSE55098	mRNA	10	12	GPL570	PBMC
GSE156035	mRNA	20	20	GPL20844	PBMC
GSE55099	miRNA	10	12	GPL8786	PBMC

*GEO* gene expression omnibus, *PBMC* peripheral blood mononuclear cell, *T1D* type 1 diabetes.

### WGCNA analysis

The adjusted microarray was analyzed using the R WGCNA package [13, 33]. The top 25% of the maximum expression variance was selected for WGCNA analysis, and 4895 genes remained for WGCNA analysis. The hclust function was used for outlier detection using “average”. The soft threshold was set to the value recommendedby the WGCNA analysis. A one-step network construction was used to construct the gene module. Then, the similarity matrix was calculated based on the eigengenes of each module using the R cor function, hclust function, and Pearson method. Clustering analysis was performed using “average” and the modules with high similarity were integrated during which 0.7 was set as the cut height. Then, the correlation analysis between the integrated module and trait was determined based on the eigengenes using the R cor function and corP valueStudent function. Subsequently, the genes in candidate modules were extracted, and based on eigengenes, the correlation between genes and modules was analyzed using the R cor function and corPvalueStudent function. Genes with a correlation coefficient better than 0.5 were selected for further analysis.

### Differential analysis

Modules with significant correlation with T1D were selected, and their correlations were analyzed using the cor function with the module eigengene being used for calculation. Genes with a correlation coefficient above 0.5 were selected as candidate genes for subsequent differential analysis. The R limma package was used to analyze the expression of candidate genes in integrated data, and genes with *p* < 0.05 were defined as differentially expressed genes (DEGs). Differentially expressed miRNAs were searched in the miRNA microarray using the Limma package based on the criteria of |logFC| > 0.7 and *p* < 0.05.

### Gene ontology (GO) and Kyoto Encyclopedia of genes and genomes (KEGG) enrichment analyses

GO and KEGG enrichment analyses were performed to detect biological functions and potential pathways of DEGs using the R clusterprofiler package with *p* < 0.05 considered as the cutoff value [34, 35].

### miRNA–mRNA network

Funrich v3.1.3 software [36] was used to predict miRNA target genes. The data of target genes and T1D-related DEGs were analyzed for overlaps, in which the target genes with reversed miRNA expression patterns were obtained. The miRNA–mRNA network was constructed using cytoscapev3.9.1 [37].

## Results

### Outlier detection

**Fig. 1:**
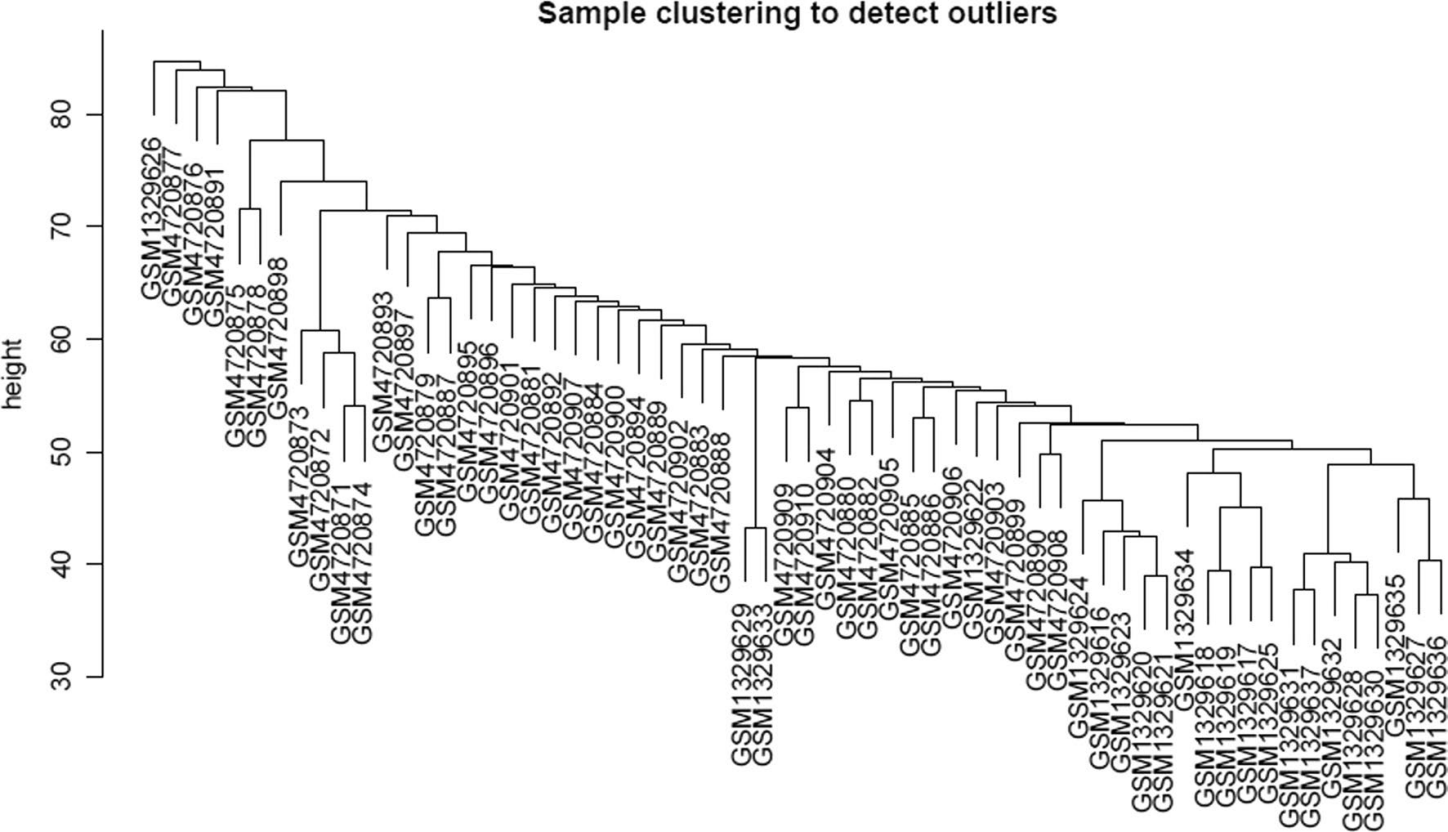
Sample clustering to detect outliers using WGCNA analysis. Note: WGCNA, weighted correlation network analysis.

Two independent T1D microarrays (GSE156035 and GSE55098) were downloaded from the GEO database. The data in these two microarrays were integrated and adjusted, and 62 samples were identified, including 30 cases of healthy controls and 32 cases of T1D. There were 19,579 coexpressed genes in the two microarrays (Additional file 1: Table S1). Then, sample clusters were detected for outliers (Fig. 1), and the detection showed no obvious outliers; therefore, all samples were included for subsequent analysis.

### WGCNA analysis

**Fig. 2:**
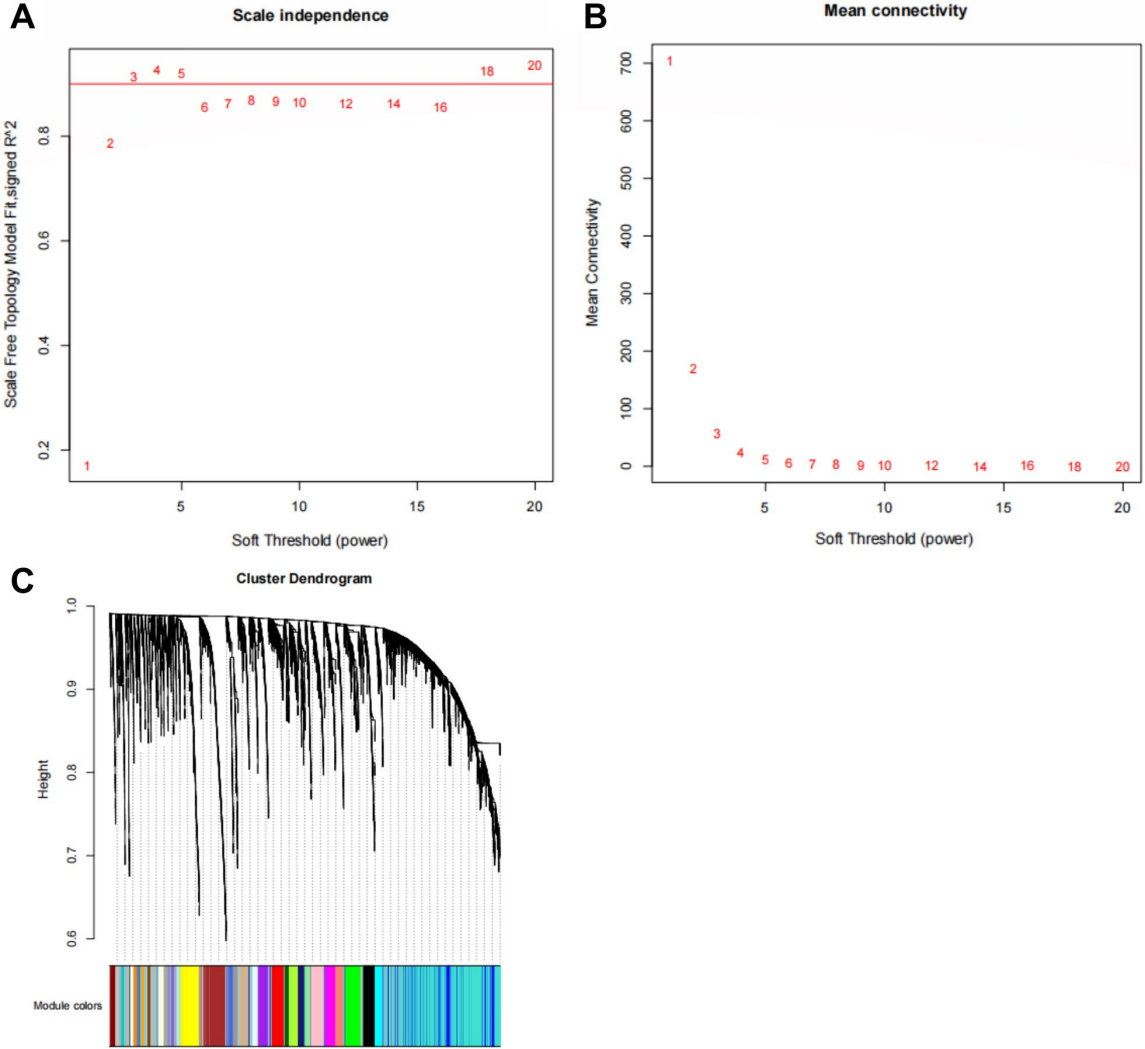
WGCNA analysis. Note: **A**, **B** The scale free topology model fit under different soft thresholding power. The number herein is the relative soft thresholding power. When soft thresholding was set at 3, the approximate scale free topology can be obtained, indicating genes can be efficiently distinguished at the soft thresholding of 3. **C** Each branch of the dendrogram stands for a gene. Based on the topology overlapped cluster, gens were classified into different modules in different color. Each color stands for one module, in which highly connected genes were included. A total of 31 modules were identified. WGCNA, weighted correlation network analysis.

Genes were analyzed using the WGCNA method, and modules related to T1D were obtained. To ascertain the obtained modules, they were screened using soft thresholding power (Fig. 2A, B). Screening showed that the scale-free parameter was approximately 0.9 in response to a soft thresholding power of 3. Therefore, the soft thresholding power was set to 3 for subsequent analysis. Genes were analyzed using a one-step method to distinguish them into 31 modules. Each module was marked in a different color (Fig. 2C), and the module in gray indicates non-significant genes.

### Integration of similar modules

**Fig. 3:**
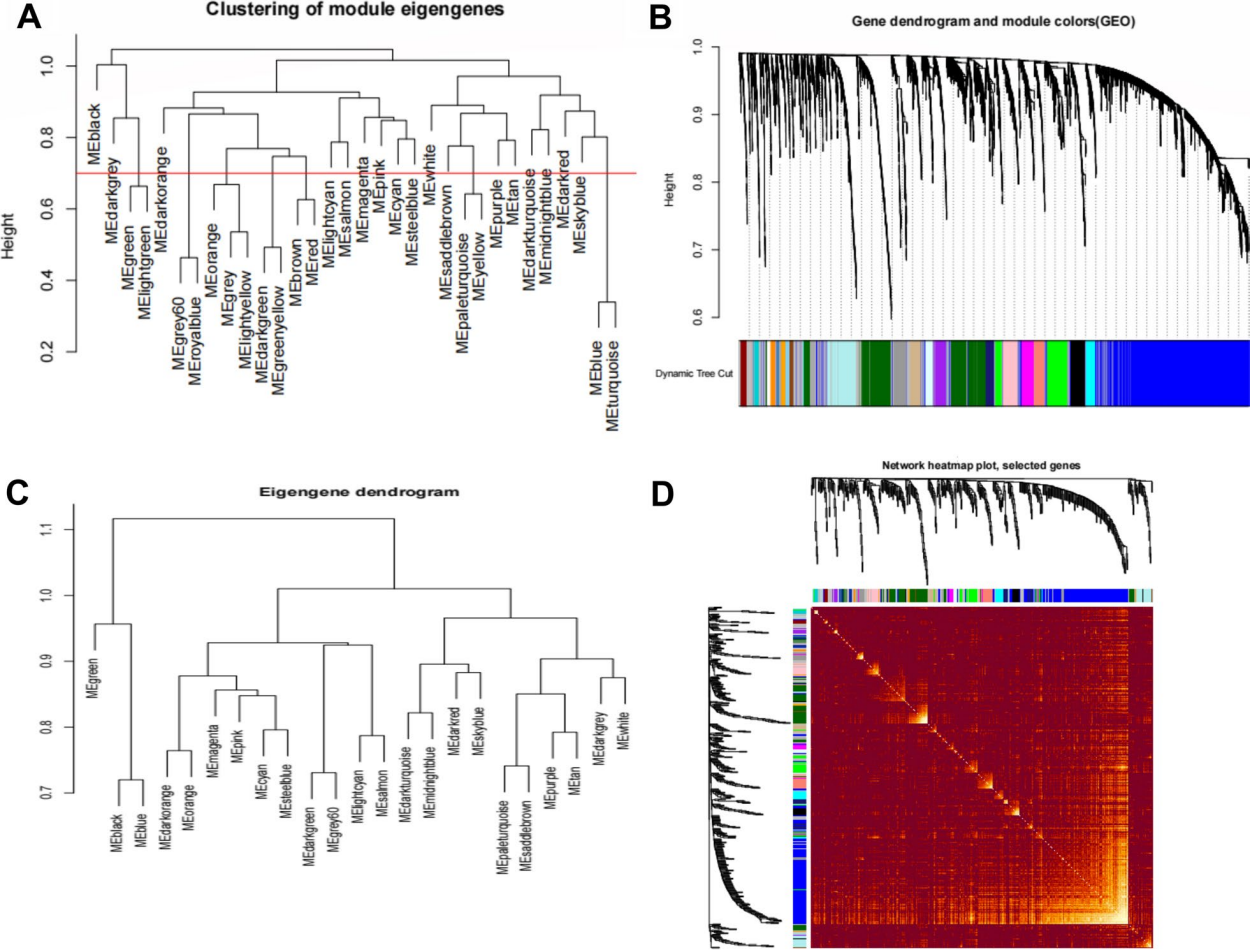
Integration of similar modules. Note: **A** Correlation analysis on modules before integration. The vertical ordinate is the similarity of modules. Lower the ordinate value, higher the similarity. Red line is the cut height. Cut height at 0.7 was set in this study and modules with cut height under 0.7 were selected for integration. **B** Gene dendrogram. **C** Module dendrogram after integration. **D** Visualization on gene networks. About 500 genes in the gene network were randomly selected and the TOM value was described by heat map.

A total of 31 modules were identified. The similarity among the 31 modules was calculated, and those with a similarity coefficient within 0.7 were integrated (Fig. 3A). A total of 24 modules were obtained, as shown in Fig. 3B, C. A total of 500 genes were randomly selected to visualize module membership (Fig. 3D).

### Correlation analysis among modules

**Fig. 4:**
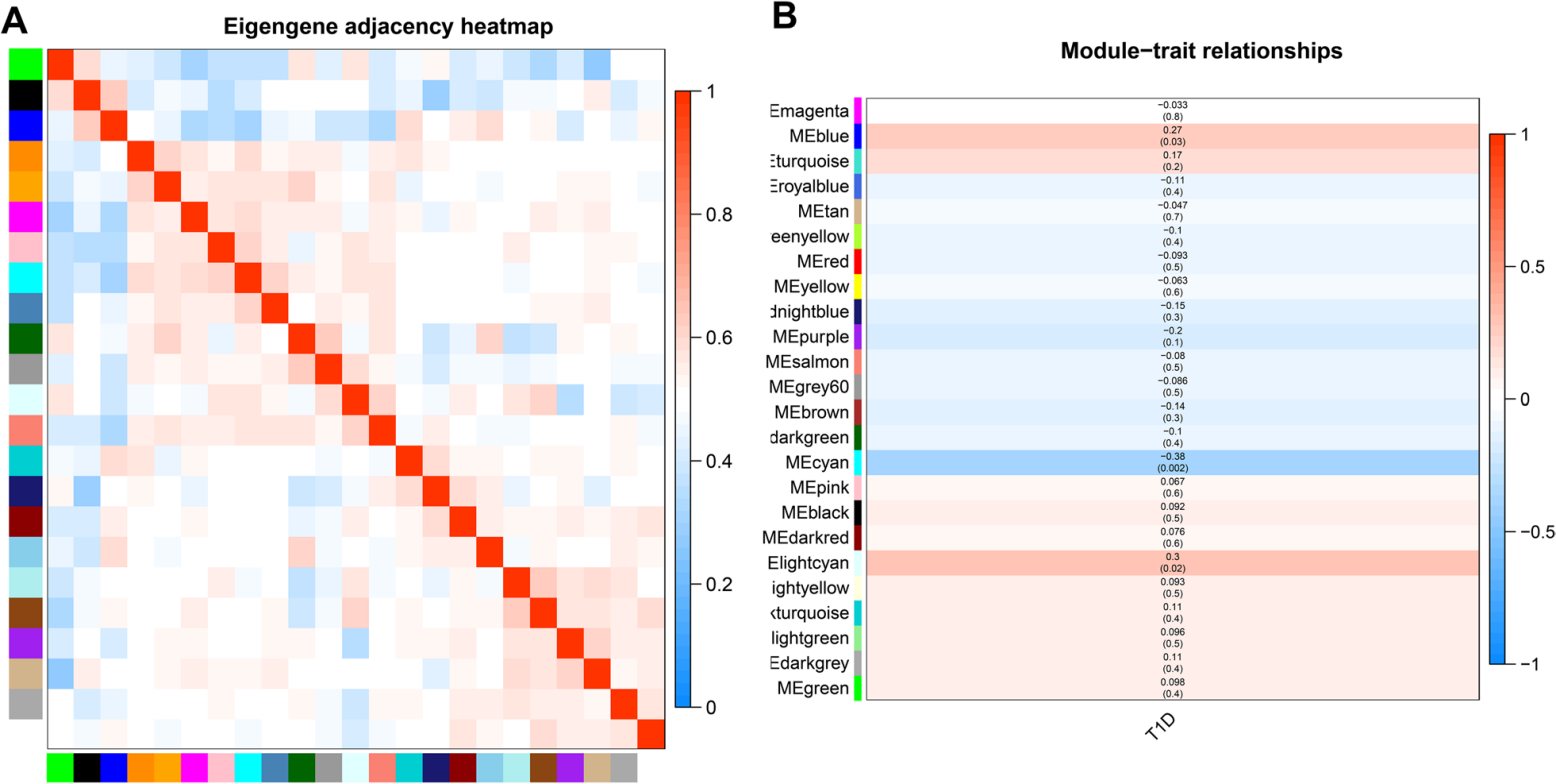
Correlation analysis among modules. Note: **A** Heat map describing the correlation among modules. Red indicates for positively correlated and blue for positively correlated. The horizontal and vertical axis indicates for different colored modules. The histogram in the right stands for color level. **B** The correlation between modules and T1D development. Numbers are the Pearson coefficient and the p value of correlation is in the brackets. The histogram in the right stands for color level.

The correlations among the 24 modules were analyzed (Fig. 4A), and the results showed no significant correlations among the modules, indicating the independence of the genome in these modules. Subsequently, the features of the T1D cases were compared with those of the 24 modules using correlation analysis (Fig. 4B). The results showed that the cyan module was negatively correlated with T1D, whereas the blue and light cyan modules were positively correlated with T1D. These findings suggest that the genes in these three modules may be of great importance in the initiation and development of T1D.

### Differential analysis of the candidate genes

**Fig. 5:**
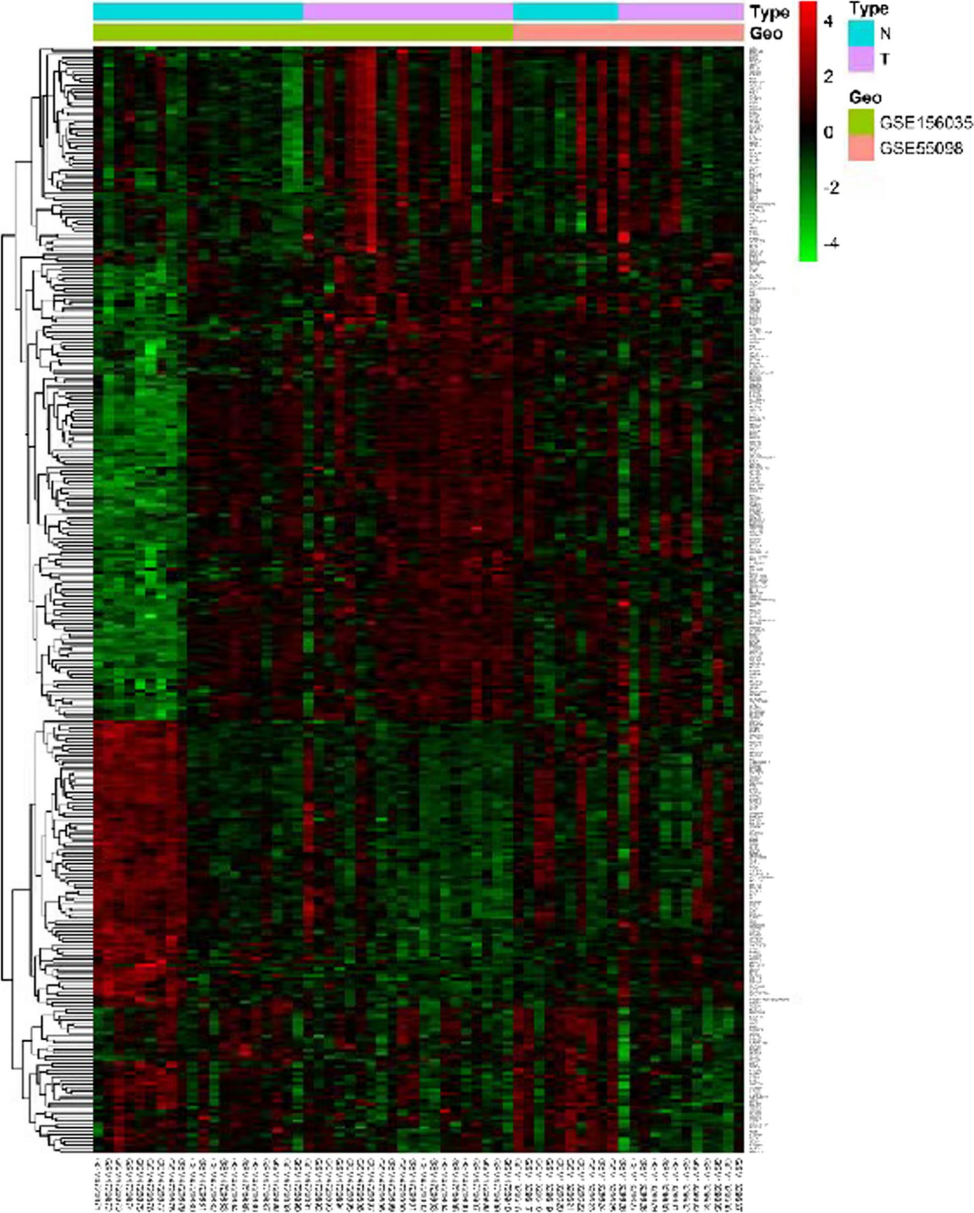
Heat map describing the expression difference of candidate genes. The horizontal axis is the sample code and vertical axis is the gene name. Dendrogram in the left is the cluster of gene expression levels. The histogram in the right stands for color level.

Genes in the above three modules were extracted, and the correlation between clustered genes and modules was calculated (Additional file 2: Table S2, Additional file 3: Table S3, Additional file 4: Table S4). Genes correlated with module eigengenes (correlation coefficient > 0.5) were selected for subsequent analysis and 926 genes were obtained (Additional file 5: Table S5). The expression differences of these 926 genes in the integrated microarray were analyzed, and 327 genes in T1D samples showed significantly different expression compared to their expression in controls (Additional file 6: Table S6 and Fig. 5). These results further support the hypothesis that these 327 genes play key roles in T1D.

### Enrichment analysis

**Fig. 6:**
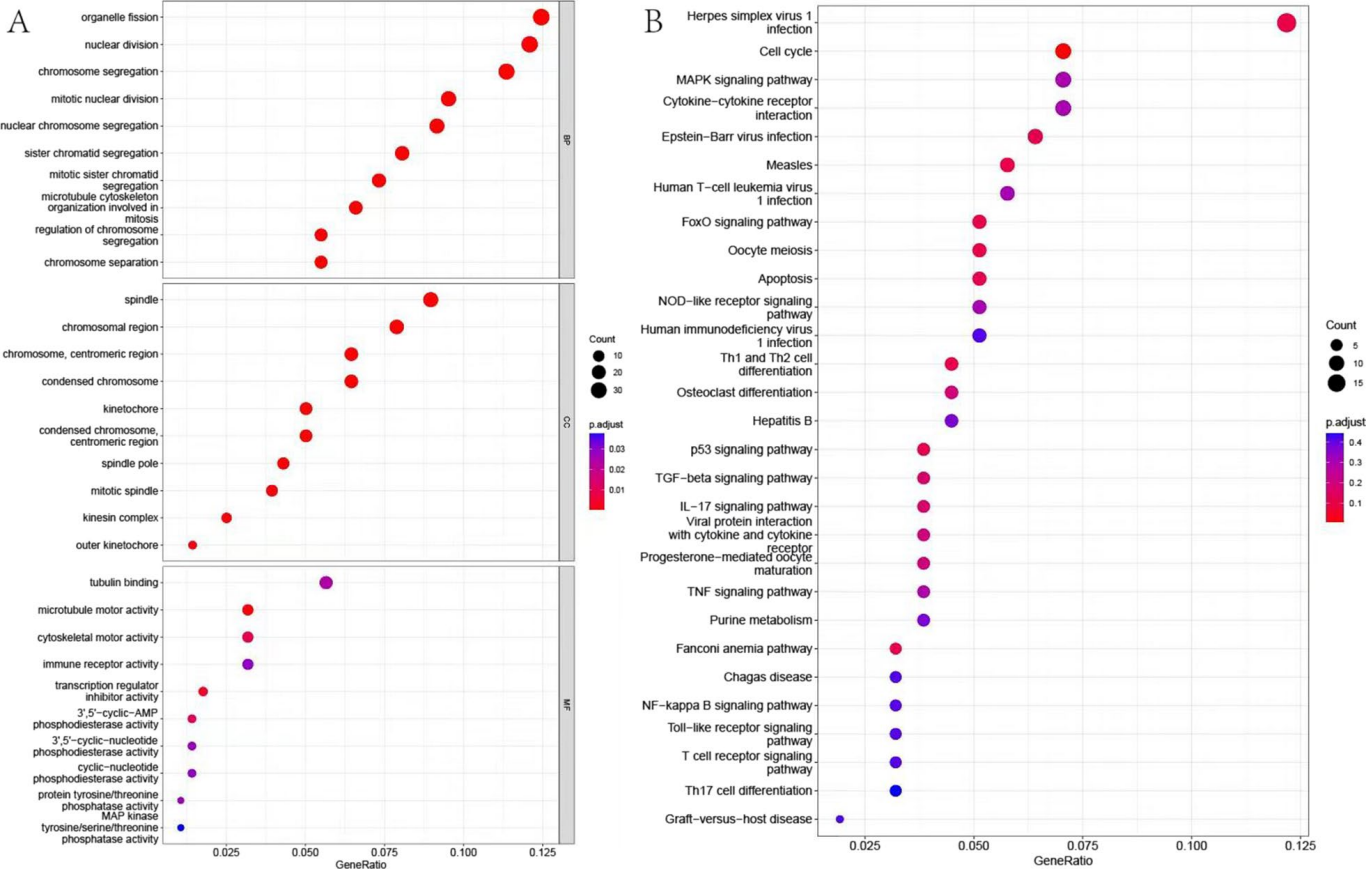
Functional enrichment analysis of candidate genes. Note: **A** GO enrichment analysis. Horizontal axis is GeneRatio and the vertical axis is category. The histogram in the right stands for color level. **B** KEGG pathway enrichment analysis. GO, Gene Ontology; KEGG, Kyoto Encyclopedia of Genes and Genomes.

The screened 327 genes were subjected to GO and KEGG pathway enrichment analysis [38–40]. GO enrichment analysis revealed that 327 genes were mainly distributed in functional categories, including HSV1 infection, cell split, and nuclear split (Fig. 6A). KEGG enrichment analysis showed that the cell cycle and MAPK signaling pathways were enriched (Fig. 6B). Studies have highlighted that T1D can lead to beta cell loss and influence the splitting of beta cells [41–43]. Additionally, the MAPK gene is involved in the initiation and development of T1D [44]. HSV1 infection has also been reported to influence T1D progression [45]. This evidence suggests that the cell cycle and MAPK signaling pathways enriched by KEGG may have an important regulatory role in T1D.

### MiRNA differential analysis

**Fig. 7:**
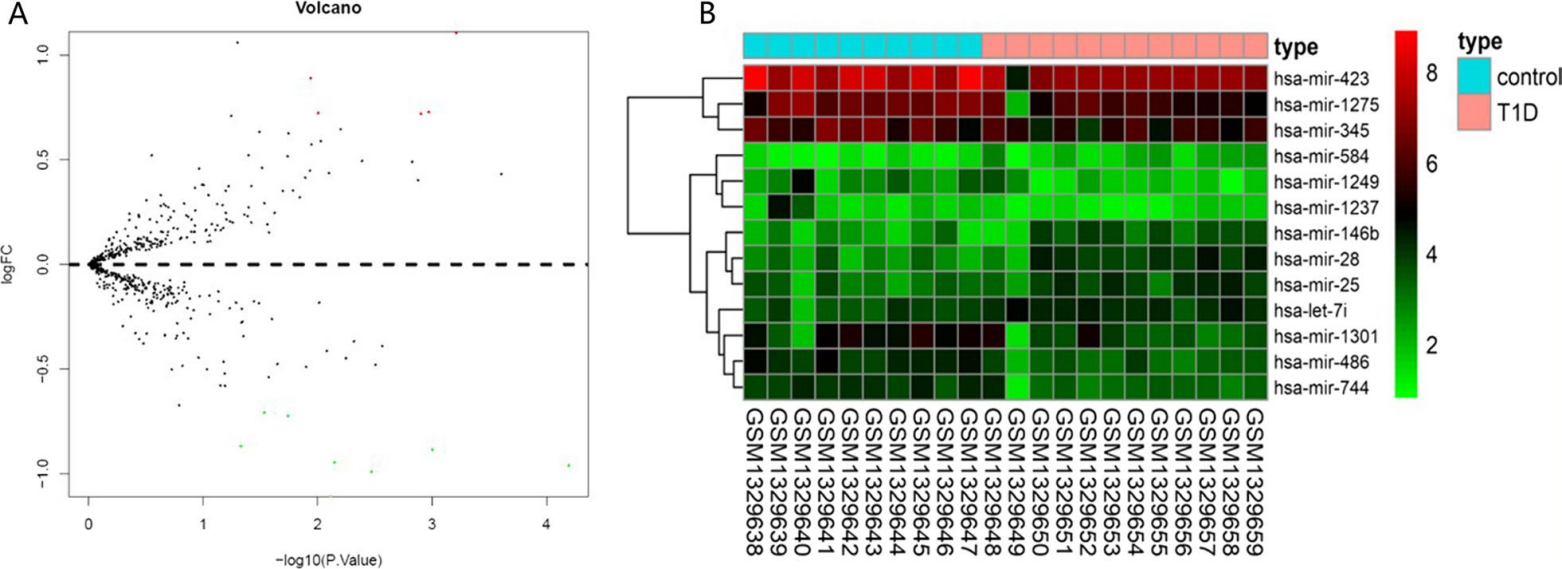
Differential analysis on T1D related miRNA microarray. Note: **A** Volcano map describing differential expressed miRNA. Horizontal axis is -log10pvalue and the vertical axis is logFC. Red dot is the miRNA that is upregulated in T1D and green dot is the miRNA that is downregulated in T1D. **B** Heap map for differential expressed miRNA. T1D, type 1 diabetes.

One independent miRNA microarray, GSE55099, was downloaded from the GEO database, which included 10 control samples and 12 T1D samples. Differential analysis on GSE55099 found 13 miRNAs with significant differential expression (Fig. 7A, B), among which some miRNAs including miR-423, let-7i, and miR-25 were reported to regulate T1D [24, 27, 46, 47]. Among the 13 miRNAs, 5 were upregulated in T1D and 8 were downregulated in T1D.

### Prediction of miRNA–mRNA network

**Fig. 8:**
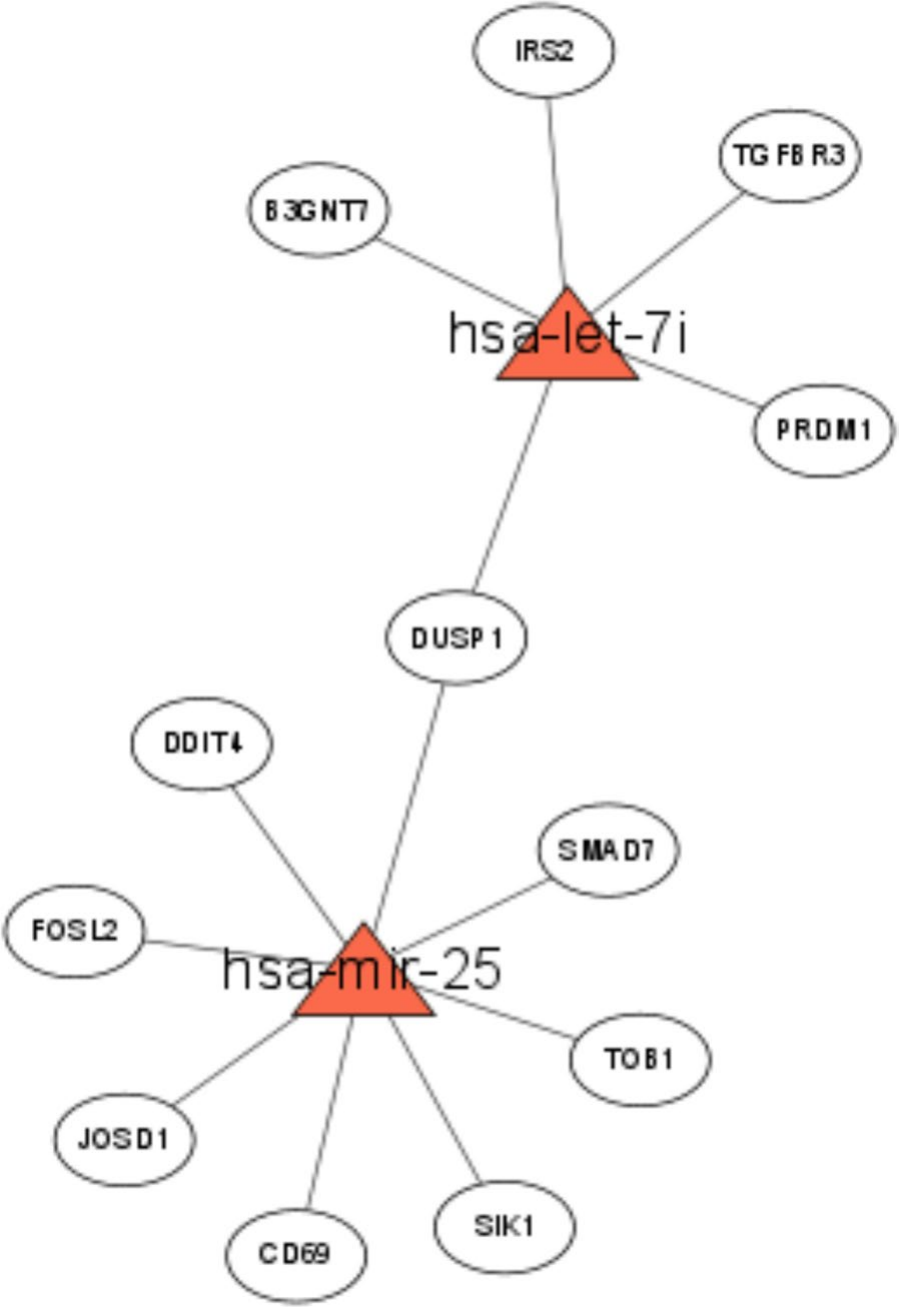
miRNA–mRNA regulatory network. Triangle indicates the candidate miRNA and circle is the candidate mRNA. The line between triangle and circle indicating the possible regulatory role between miRNA and mRNA.

The target genes of the 13 candidate miRNAs were predicted (Additional file 7: Table S7). The results of the differential expression of miRNAs in T1D and the prediction of their target genes were analyzed for overlaps with the 327 genes to screen the genes that had reversed expression with the candidate miRNAs. Subsequently, an miRNA–mRNA regulatory network was established. Among the 13 miRNAs, 2 miRNAs were identified with their target genes with significantly differential expression in T1D (Fig. 8). These results suggest that these two miRNAs may play key roles in regulating T1D by mediating different target genes.

### Conclusion

Multiple T1D microarrays were integrated to obtain the T1D-related gene expression data. The gene expression data were screened using WGCNA and differential analysis, and 327 genes were identified to have a close relationship with T1D. In parallel, one miRNA microarray related to T1D was downloaded for analysis and 13 candidate miRNAs were identified to be associated with T1D. Further prediction of the 13 miRNAs and correlation analysis with the 327 genes contributed to the miRNA–mRNA regulatory network. Two miRNAs and 12 mRNAs had significant differential expression in T1D. These results suggest that miRNAs and mRNAs may play certain roles in the initiation and development of T1D.

## Discussion

Network-based analysis is an effective approach for exploring the connection between genes and pathways in diseases. This study used WGCNA analysis to integrate the clinical features of T1D patients with genome profiles to classify genes into several modules. Next, the correlation of modules with T1D was conducted to screen the key genes involved in T1D initiation and development.

WGCNA analysis generated three modules that were closely associated with T1D, and the genes in the modules were extracted for correlation analysis. Finally, 926 candidate genes were identified, in which differential expression analysis of these candidate genes yielded 327 genes. GO enrichment of these genes showed that they were involved in the cell-splittingprocess. In T1D, the number of beta cells decreases with the progression of T1D [43]. Nakanishi et al*.* identified that beta cells were decreased by 90% in T1D cases compared with controls [48]. Similarly, failure to detect beta cells in T1D was reported by Butler [49], and Diedisheim et al. detected no beta cell mass in seven T1D cases with disease progression of 5–25 years [50]. A report on 54 cases of T1D by Campbell-Thompson also failed to detect beta cell mass in the majority of T1D samples [42]. GO analysis and the above reports suggest that split beta cells may be of great importance in the development of T1D. KEGG enrichment analysis further identified that the 327 genes were mainly involved in the MAPK signaling pathway. In agreement with our results, the MAPK signaling pathway was shown to have a close relationship with T1D [51, 52].

To locate the key genes involved in T1D progression, we screened an miRNA microarray in the GEO database, based on which differential analysis was conducted, and 13 miRNAs were obtained. Then, the target genes of these 13 miRNAs were predicted, and further screening of the expression pattern of miRNAs and target genes contributed to the establishment of the miRNA–mRNA network. Eventually, 2 miRNAs and 12 target genes were identified. Consistently, let-7i has been implicated in T1D regulation [27], and miR-25 is believed to be associated with the function of beta cells in T1D [24]. The above results suggest that the candidate miRNAs may directly regulate T1D progression; however, how they regulate T1D development has rarely been reported. In this study, a total of 12 genes were screened, including *B3GNT7*, *IRS2*, *TGFBR3*, *PRDM1*, *DUSP1*, *SMAD7*, *TOB1*, *SIK1*, *CD69*, *JOSD1*, *FOSL2*, and *DDIT4* by WGCNA analysis, and may be regulated by let-7i and miR-25. Among these genes, *DUSP1* and *SMAD7* have been reported to be involved in the regulation of T1D [53–57]. In addition, *IRS2* is believed to be involved in the functional regulation of INS-1 cells to mediate T1D development [58]. In addition, a close relationship between *IRS2* and insulin-related pathways has been documented in previous studies [59, 60]. A Chinese study reported that *SMAD7* was implicated in the regulatory effect of the compound Coptodis Rhizoma capsule in diabetic rats [61]. Despite these studies, some candidate genes have not been reported in T1D, such as *SIK1*, *FOSL2*, and *DDIT4*. However, these genes have been found to regulate type 2 diabetes [62–66], suggesting that these 12 candidate genes may have an important regulatory role in T1D. Comprehensive research on these genes will facilitate our understanding of the T1D pathogenesis.

In this study, we identified 2 miRNAs and 12 mRNAs that may have certain effects on the regulation of T1D. Based on this analysis, it was noted that most of the mRNAs and miRNAs were not reported in T1D, which provides new research directions for T1D pathogenesis. In addition, one of the highlights of this study is to conduct further analysis of a possible regulatory mechanism based on the candidate genes. Overall, through WGCNA analysis, this study was conducted to facilitate our understanding of genes and mechanisms related to T1D progression.

## Supplementary Information

**Additional file 1.** Two independent microarrays related to T1D (GSE156035 and GSE55098) were integrated for analysis and 19579 co-expressed genes were identified in both microarrays.

**Additional file 2.** The genes in the module 1 were extracted and the correlation of clustered genes with modules was calculated.

**Additional file 3.** The genes in the module 2 were extracted and the correlation of clustered genes with modules was calculated.

**Additional file 4.** The genes in the module 3 were extracted and the correlation of clustered genes with modules was calculated.

**Additional file 5.** Genes with a correlation coefficient above 0.5 were selected for subsequent analysis and 925 candidate genes were obtained.

**Additional file 6.** The 925 candidate genes were used for differential analysis and 327 genes were found to have significant differences.

**Additional file 7.** Prediction on the target genes of 13 candidate miRNAs (hsa-mir-28, hsa-mir-744, hsa-let-7i, hsa-mir-584, hsa-mir-423, hsa-mir-1249, hsa-mir-1275, hsa-mir-25, hsa-mir-146b, hsa-mir-345, hsa-mir-1237 and hsa-mir-1301.

## Data Availability

The datasets generated and/or analyzed during the current study are available in the Gene Expression Omnibus (GEO) repository (https://www.ncbi.nlm.nih.gov/geo/).
